# Health Professionals’ Views on Euthanasia: Impact of Traits, Religiosity, Death Perceptions, and Empathy

**DOI:** 10.3390/healthcare13141731

**Published:** 2025-07-18

**Authors:** Dimitrios Mimarakis, Maria Moudatsou, Philippa Kolokotroni, Athanasios Alegakis, Sofia Koukouli

**Affiliations:** 1 Department of Social Work, Faculty of Health Sciences, Hellenic Mediterranean University, 71410 Heraklion, Greece; moudatsoum@hmu.gr (M.M.); koukouli@hmu.gr (S.K.); 2 Laboratory of Interdisciplinary Approaches for the Enhancement of Quality of Life (QoLab), 71410 Heraklion, Greece; 3 Social Service, Pammakaristos Hospital of Divine Providence, 11144 Athens, Greece; 4 Department of Psychology, Panteion University of Social & Political Sciences, 17671 Athens, Greece; philippakolokot@gmail.com; 5 Department of Toxicology, Faculty of Medicine, University of Crete, 71003 Heraklion, Greece; alegkaka@uoc.gr

**Keywords:** euthanasia, end-of-life, empathy, health professionals, attitudes

## Abstract

Context: A plethora of actors like individual and professional characteristics, religiosity, personality-related factors, personal experience of death, and empathy form the attitude of health professionals in patient care in clinical settings and euthanasia. Objectives: The aim of the study was to measure the attitudes of health professionals toward euthanasia. In addition, we examined how individual and professional characteristics, religiosity, death perspectives, and empathy may predict health professionals’ attitudes toward euthanasia. Methods: We collected socio-demographic characteristics and attitudes toward euthanasia and the end of life as well as empathy dimensions from 465 health professionals working in health services in Greece. Multiple linear regression was applied to test the association of the attitudes about euthanasia with (1) demographic and professional variables, (2) perceptions about death, and (3) empathy. Results: Findings of the study revealed that health professionals’ attitudes towards euthanasia are significantly associated with their age, the target group of their patients, religious beliefs, and their attitudes about the end of life. Meanwhile, empathy was an insignificant predictor of ATE (*p* > 0.05). Conclusions: Working in clinical settings with patients, especially at the end of their lives, will always include ethical issues for health professionals. Training and supporting new generations of health professionals in patient care and bioethics are crucial in order to face these ethical issues during their professional careers.

## 1. Introduction

Euthanasia has been a subject of research and ongoing debate among scholars and the academic community for several decades. The concept of euthanasia is frequently the subject of media controversy, influenced by social, cultural, and religious factors [1]. Numerous international studies have demonstrated that attitudes toward euthanasia are shaped by the sociocultural context, religious beliefs, professional background, age, and the diversity of perspectives and that these attitudes evolve over time [2]. According to the findings of Butt, Overholser, and Danielson [3], factors such as age, race, levels of hopelessness, and various psychological characteristics among college students may influence an individual’s acceptance of euthanasia and physician-assisted suicide (PAS) [3].

It has been emphasized on multiple occasions that PAS refers to instances in which a physician provides a patient with the means—typically lethal medication—to end their own life. Furthermore, among 221 healthcare professionals at Yale-New Haven Hospital who supported the legalization of physician-assisted death (PAD), justifications included relief of suffering, the right to die, mercy, acceptance of death, non-abandonment, and the potential for reducing healthcare costs [4].

In this study, we examined for the first time in Greece the association of individual and professional characteristics, perceptions about death, and empathy with attitudes toward euthanasia in health professionals. We focused on health professionals’ attitudes toward euthanasia because (a) euthanasia has been a research subject and a matter of debate among scientists, health professionals, and the general population for several decades, (b) criminal law in Greece has no special regulations justifying or even allowing any act of euthanasia, and (c) the vast majority of people are against euthanasia as a result of tradition and religion [5].

### 1.1. Literature Review

#### 1.1.1. Euthanasia

Euthanasia and assisted suicide have been subjects of ongoing historical discourse, primarily because they relate to the broader concept of the right to life—a principle that has received sustained support over time [6].

Although assisted suicide differs from euthanasia in that the patient makes the ultimate decision, the terms “assisted death” can be used to refer to both procedures [7]. Specifically, euthanasia can be defined as the intentional act by one individual to cause the death of another, employing the most humane and painless methods, with the sole motivation of serving the best interests of the person who dies [8].

Euthanasia may be classified as either active or passive and as voluntary or involuntary. Voluntary euthanasia involves the explicit consent of the patient to end their life, while involuntary euthanasia occurs without the patient’s consent. Active euthanasia refers to the deliberate act of ending a life through the administration of a substance, such as a lethal drug, whereas passive euthanasia involves the withdrawal or withholding of medical treatments that could potentially prolong life [9]. Globally, active euthanasia is legally permitted in seven countries, with the most notable examples including the Netherlands, Belgium, Luxembourg, Canada, and Spain [10]. Physician-assisted suicide (PAS) is legalized in countries such as Germany and Switzerland, as well as in several U.S. states, including Oregon, California, Washington, and the District of Columbia [10].

In Greece the vast majority of people are against euthanasia as a result of tradition and religion [5]. Certain forms of euthanasia appear to occur covertly or “behind closed doors,” highlighting the perceived urgency to address existing legal ambiguities [11]. Furthermore, Christianity and Islam explicitly prohibit euthanasia, as does Judaism. In general, the Abrahamic religions collectively oppose all forms of assisted death, including active and passive euthanasia, as well as physician-assisted suicide [12].

Over time, numerous arguments have been presented both in support of and against assisted dying; however, public demand for the legalization of euthanasia and assisted suicide is currently stronger than ever [13].

#### 1.1.2. End of Life

Human responses to the end of life and death are complex, multifaceted, and dynamic. Since the 1970s, it has been widely held that fear of death is a universal human experience, and the absence of such fear is often interpreted as a form of death denial [14]. Mikulincer and Florian [15] outline three dimensions of death fear: intrapersonal, interpersonal, and transpersonal concerns related to death. In contrast, Kübler-Ross [16] viewed acceptance as the final stage of dying. Klug and Sinha [17] define death acceptance as “being relatively comfortable with one’s awareness of personal mortality,” highlighting the conscious acknowledgment of mortality accompanied by a positive emotional response to this awareness. Thus, death acceptance comprises two elements: recognizing one’s finite nature cognitively and reacting positively (or at least neutrally) to this understanding [14]. Approach acceptance is grounded in transpersonal religious or spiritual beliefs in a favorable afterlife. Harding, Flannelly, Weaver, and Costa [18] found that belief in God’s existence and the afterlife were both negatively correlated with death anxiety and positively associated with death acceptance.

Medical education and training on the perception of death, particularly a dignified death, appear to be crucial for understanding the necessity of creating medical–legal instruments that safeguard human dignity until the end of life [19,20]. End-of-life care will continue to be a subject of debate due to the inherent tensions among biomedical principles, diverse legal frameworks, and the beliefs held by the general population [21].

#### 1.1.3. Empathy

Empathy is a fundamental concept within psychodynamic, behavioral, and person-centered approaches due to its central role in fostering the therapeutic relationship with clients [22,23], thereby serving as a foundation for therapeutic change [22]. Empathy is the capacity to perceive and comprehend another person’s emotional state and thoughts by imaginatively placing oneself in their position and temporarily experiencing their perspective [24,25]. Rogers [26] conceptualizes empathy as the ability to perceive the client’s inner experience as if it were one’s own, while maintaining an awareness of the “as if” distinction [27].

Based on an extensive review of relevant literature, we define empathy in patient care as “a primarily cognitive attribute that encompasses the understanding of the patient’s experiences, concerns, and perspectives, along with the ability to effectively communicate this understanding and the intention to provide assistance” [28,29,30]. A substantial proportion of health professionals—approximately 70%—report difficulties in developing empathy toward their patients [27,31]. Several factors have been identified as barriers to the cultivation of empathy, including high patient loads, insufficient time, an academic culture predominantly focused on therapeutic interventions, and a lack of formal education and training in empathy [23]. Conversely, self-esteem, work engagement, and emotional regulation have been found to be positively associated with empathy [32,33]. Furthermore, several studies suggest that female gender is correlated with higher levels of empathy [34,35]. Age, self-reflection, emotional appraisal, and the expression of emotions have been specifically associated with empathy among female social workers [23]. Moreover, empathy has been positively correlated with both reflective capacity and emotional intelligence in practicing social workers as well as in social work students [36,37]. The significance of empathy is well established, as health professionals who exhibit higher levels of empathy tend to perform more effectively in facilitating therapeutic change [23].

Empathy encompasses multiple aspects. It consists of both emotional and cognitive empathy [38]. Emotional empathy occurs when someone witnesses another person’s emotional condition [39]. The cognitive dimension involves interpersonal sensitivity and understanding of others’ perspectives [23]. Cognitive empathy can approach other people’s situations in two different ways by making use of imagining her perspective and by imagining another’s perspective [39]. Healthcare professionals who try to understand other people’s suffering and pain and desire for euthanasia judge by the cognitive empathy approach. When they imagine by their perspective there are many factors such as their personal and social norms, religious beliefs, education, and their social and contextual attitudes that might influence their judgment about others’ demand for euthanasia [39].

## 2. Materials and Methods

### 2.1. Study Design

This is a quantitative study with cross-sectional design carried out in a purposive sample of healthcare professionals.

### 2.2. Participants

A large sample of Greek health professionals who were working in various clinical settings participated in the study. Data collection was performed via a Google Forms questionnaire, consisting of four parts and which remained online for a period of eight months. In order to avoid methodological sampling bias in the study, the Google Forms questionnaire was distributed across various institutions, targeting diverse medical, nursing, and other healthcare specialties in different geographic regions of Greece. The networking channels utilized included email contacts of professionals affiliated with recognized and officially established associations and organizations. Most of the participants were recruited via the members’ bulletin of the Medical Association of Athens and the Greek Association of Social Workers as well as social media. The final sample, with complete valid questionnaires, consisted of 465 health professionals. It is worth mentioning that there was no missing data.

### 2.3. Measures

#### 2.3.1. Participants’ Socio-Demographic Profile

The first part of the questionnaire consisted of participants’ self-reported socio-demographic and professional characteristics.

#### 2.3.2. Euthanasia Attitude Scale

The ATE scale [40,41] is a ten-item instrument designed to assess attitudes toward euthanasia, developed by Wasserman, Claire, and Ritchie in 2005. The Cronbach’s alpha for the original scale was 0.87 while the value 0.91 was estimated in our survey. This is a 5-point Likert scale from strongly disagree (1) to strongly agree (5). Two items were reversed coded to prevent response bias from the participants [1]. The original factorial structure was composed of four dimensions: severe pain, no recovery, patient request, and doctor’s authority [1]. The summary scores of the ATE scale result in a maximum score of 38 (with 38 = 38 and above). The Greek version of the ATE scale has undergone comprehensive psychometric validation by Mimarakis and colleagues. The manuscript is presently under review in a peer-reviewed scientific journal and is anticipated to be published in the foreseeable future. Findings from this study indicate that the ATE scale constitutes a reliable and valid instrument for assessing attitudes toward euthanasia among Greek healthcare professionals.

#### 2.3.3. Death Attitude Profile Scale

The Death Attitude Profile-Revised (DAP-R) [42] is a 32-item, Likert-scaled measure of death acceptance and avoidance as well as fear of death. The Cronbach’s alpha was estimated at 0.77 for the DAP-R scale in our survey. The DAP-R is a revision of the Death Attitude Profile (DAP) [43]. The DAP-R consists of five factorially derived dimensions: Approach Acceptance (10 items), Fear of Death (7 items), Death Avoidance (5 items), Escape Acceptance (5 items), Neutral Acceptance (5 items). For each DAP-R dimension, a mean scale score can be computed by dividing the total scale score by the number of items comprising each scale.

#### 2.3.4. Scale of Empathy

The Jefferson Scale of Empathy (HP version)] [29,30,31,32,33,34,35,36,37,38,39,40,41,42,43,44] is a widely utilized instrument developed by researchers at Thomas Jefferson University to assess empathy in healthcare professionals. The Cronbach’s alpha coefficient for JSE was estimated at 0.80. The scale employs a seven-point Likert format to capture varying degrees of empathic engagement (1 = strongly disagree; 7 = strongly agree) to measure empathy in physicians and other health professionals involved in patient care in a clinical setting. The JSE is intended to promote empathy, a vital component in delivering compassionate care, improving patient outcomes, and advancing patient-centered healthcare practices. The total score is derived from the aggregate of all individual item scores on the scale. Higher scores reflect a greater degree of empathic behavioral orientation.

### 2.4. Statistical Methods

Mean and standard deviation were used to describe the continuous variables such as questionnaire scores, while discrete data were presented in the form of frequencies and %frequencies. Normality tests (Kolmogorov–Smirnov) and measures of internal consistency (Cronbach’s alpha) were used for continuous variables. Based on normality results, parametric (independent samples *t*-test, one-way ANOVA) or non-parametric (Mann–Whitney, Kruskal–Wallis) tests were used interchangeably. Multiple linear regression was applied to test the association of the attitudes about euthanasia vs. empathy (JSE scale) and perceptions about death (DAP-R scales) with the presence of various demographic and occupational variables. IBM SPSS Statistics 24.0 was used for statistical analysis of data and a level of α = 0.05 was set as the level of significance.

## 3. Results

### 3.1. Participants’ Profile

In Table 1, the demographic and professional characteristics of the personnel who participated in the study are presented. Women constituted the majority of the sample (348, 74.8%). The age distribution showed a lower percentage of personnel ≤30 years old (74, 15.9%), while those >41 years old were 252 (54.2%). The mean age of the personnel is 42.6 ± 10.9 with a range from 20 to 79. Regarding marital status, 241 (51.8%) were married. Two hundred and two professionals (43.4% of the total sample) held a master’s degree or a PhD. Ninety-two persons (19.8%) have professional experience ranging from 21–30 years. Most of the participants were social workers (185, 39.8%), followed by physicians (122, 26.2%) and nurses (79, 17.0%), while the rest are other health professions like physiotherapists, pharmacists, and psychologists. In terms of employment status, 97.0% had a full-time job, 67.3% were employed in the public sector, 57.2% were working in a hospital, and 55.1% were working with end-stage patients.

**Figure 1:**
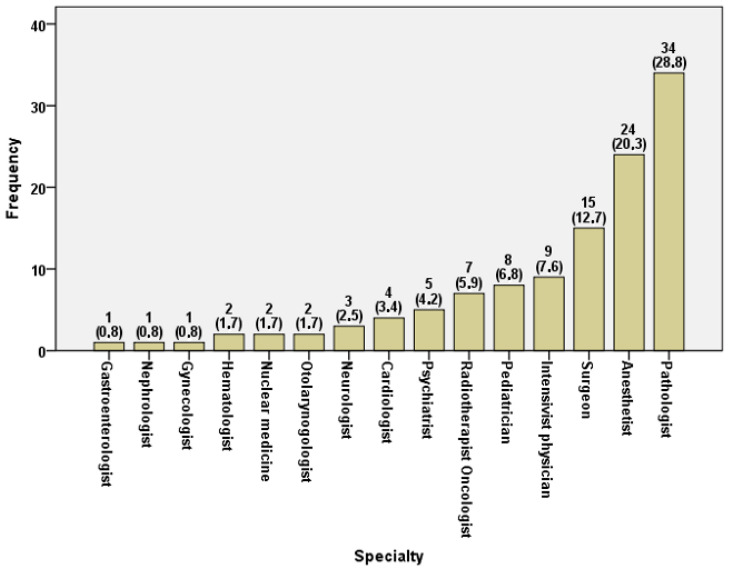
Distribution of medical specialties in the survey.

Specialties’ distribution in the physicians’ subsample showed that 34 (28.8%) were pathologists, 24 (20.3%) anesthetists, and 15 (12.7%) surgeons and all other reported specialties showed percentages lower than 10% (Figure 1).

The distribution of religiosity, shown in Figure S1 (Supplementary Material), indicates that 166 (35.7%) of the participants were “moderately religious,” 143 (30.8%) “slightly religious,” and there is a group of 86 (18.5%) participants who self-reported as “atheists.”

### 3.2. ATE Scale Analysis

Internal consistency measured using Cronbach’s alpha was excellent (a = 0.907). The ATE scale was not normally distributed (*p* < 0.001) and the mean ATE score was 24.1 ± 9.2, with a median of 24.0 and a range from 10 to 50. The scale showed a median or mean total score around 24, indicating a low acceptance of euthanasia. As shown in Table 2, there are no significant differences in the ATE scale related to the sex of the participants, marital status, educational level, and age groups.

Attitudes toward euthanasia differ significantly (*p* = 0.045) based on the specific target groups of patients with whom health professionals engage in their workplaces. Healthcare personnel working with the elderly reported the lowest mean ATE score of 20.7 ± 8.6 in comparison to other target groups (minors: 24.5 ± 8.8, adults: 25.1 ± 9.6, and combination of groups: 23.6 ± 5.5). On the other hand, no significant differences were found in ATE mean score for profession, years of professional experience, employment status, working hours and sector, physicians’ specialisms, and working with end-stage patients (Table 3).

The association of ATE vs. DAP-R and JSE is presented in the following section. There is a significant correlation of ATE scale with most of the DAP-R subscales but not with JSE. The DaV scale showed a stronger correlation (r = −0.358, *p* < 0.001) than Nac (r = −0.099, *p* = 0.032), Aac scale (r = 0.092, *p* = 0.048), and Eac scale (r = −0.173, *p* < 0.001) (Table 4).

Religiosity affected the ATE total score (*p* = 0.001). The mean score in extremely religious health professionals was the lowest (20.4 ± 9.6) compared to the moderately religious (22.4 ± 8.3), slightly religious (25.5 ± 9.1), and the atheists (26.3 ± 9.5) (*p* = 0.001). The results were similar when “Prefer to not say” (24.5 ± 10.1) was excluded (*p* < 0.001) (Figure S3, Supplementary Material).

### 3.3. Multiple Regression Models for ATE Prediction

**Figure 2:**
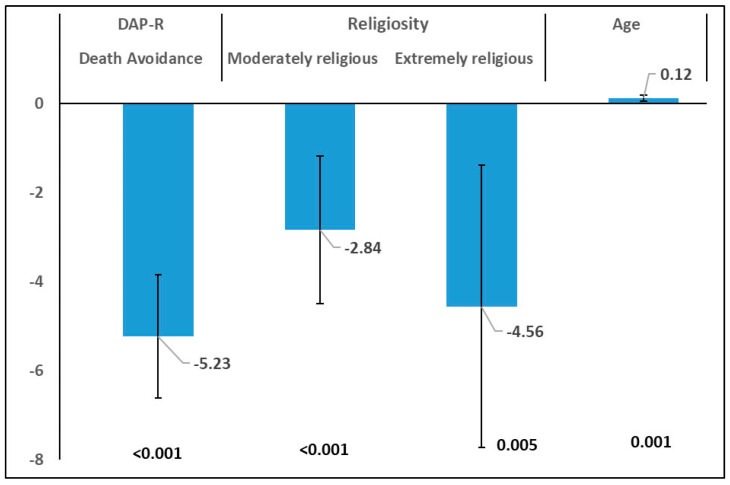
Multiple regression model of ATE vs. Personal Characteristics and DAP-R (Beta and 95% CI).

Based on previous associations between ATE and explanatory variables a multiple linear regression model was applied. All variables that showed a *p*-value for association lower than 0.200 were included in the initial model. The initial list included gender, age, target group, JSE score and DAP-R scores, and religiosity. After applying a stepwise regression technique, the final model revealed that ATE was associated with age (b = 0.12, 95%CI: 0.05–0.20, *p* = 0.001), DAP-R DaV (b = −5.23, 95%CI: −6.62 to −3.84, *p* < 0.001), moderate religiosity (b = −2.84, 95%CI: −4.50 to −1.19. *p* = 0.001), and extreme religiosity (b = −4.56, −7.73 to −1.39, *p* = 0.005) (Figure 2).

## 4. Discussion

Statistical analyses showed significant relationships of the health professionals’ attitudes toward euthanasia with religious beliefs, the target group of patients, and the age of personnel.

Several studies have shown a negative influence on most dimensions of religion to euthanasia, and in fact, it is well documented that weaker religious belief is the most important factor associated with higher acceptance of euthanasia [2,12,45,46]. A stable predictor for the current study’s health professionals’ attitudes toward euthanasia is their religious beliefs. Religiosity affected ATE total score and, specifically, ATE was associated with moderate religiosity and extreme religiosity. Similarly, the study by Velasco Sanz et al. found that religious believers were more likely to oppose the regulation of euthanasia and medically assisted suicide, as well as the recognition of an individual’s right to make autonomous decisions regarding how they wish to live and die [45]. Conversely, non-believing respondents were more supportive of shared decision making between physicians and responsible nurses in response to requests for assisted dying, as well as the administration of euthanasia by both nursing and medical professionals [45]. The findings of Sabriseilabi and Williams’ study [12], which analyzed data from 1066 adults surveyed in the 2018 General Social Survey in the United States, demonstrated that religiosity, belief in an afterlife and heaven, and religious denomination were significantly associated with opposition to euthanasia. Notably, a nationwide study conducted in the Netherlands reported that respondents with higher levels of education and no religious affiliation were more likely to support the right to make autonomous end-of-life decisions [46].

Indeed, the impact of Hippocratic philosophy and the humanistic teachings of the Christian Orthodox Church has led both physicians and the public to regard the issue of euthanasia with aversion [5]. While the Greek Orthodox Church maintains a negative stance, the government refrains from assuming the political cost associated with regulating this peripheral issue [11].

Interestingly, the target group of work was significantly associated with health professionals’ attitudes toward euthanasia. More specifically, healthcare professionals who work with elderly people showed the lowest ATE score in comparison to other target groups (e.g., minors, adults, and a combination of groups). A possible explanation for this finding could be that health professionals and care staff working with the elderly may have a more favorable view of palliative care. There is also a suggestion that the focus may shift from euthanasia to murder, with the legalization of euthanasia potentially having a significant impact on marginalized groups, including the poor, rejected, and disabled [38]. Many consider the elderly as part of these vulnerable population groups. The study by Ercan Avci concludes that alleviating a person’s unbearable pain and suffering constitutes a fundamental objective of medicine; however, this objective should be pursued through morally acceptable methods rather than euthanasia or physician-assisted suicide [47].

We also found a positive correlation between healthcare professionals’ age and their attitudes toward euthanasia. A number of studies conducted globally reveal the association of attitudes towards euthanasia with age [2]. According to the findings of Butt, Overholser, and Danielson’s study, age may influence college students’ acceptance of euthanasia and physician-assisted suicide (PAS) [3]. Specifically, we found that elder health professionals were more positioned in favor of euthanasia practice. In line with our findings, the study of Velasco Sanz et al., which aimed to examine the opinions of Spanish nurses regarding euthanasia and medically assisted suicide, found that nurses over 51 years of age were more in favor of the regulation of both euthanasia and medically assisted suicide (MAS) and of the involvement of nurses in health policy [45]. This may be attributed to the growing personal relevance of death as individuals age.

There is a significant correlation of the ATE scale with most of the DAP-R subscales (e.g., DAP-R DaV) but not with JSE. In line with our findings, the study of Stergiannis et al. [48], which aimed to examine the attitudes and perspectives of medical and nursing staff in four regional Greek hospitals about euthanasia using the Euthanasia Attitude Scale (EAS) [49], found a correlation of positive attitudes towards euthanasia with lower acceptance of death or neutral acceptance. It is worth mentioning that studies examining the associations of any euthanasia scale, especially of ATE and DAP-R, are scarce.

Despite the high mean and median scores for empathy, it was found that empathy in healthcare professionals did not significantly predict variations in attitudes toward euthanasia. While this finding may be considered unexpected, it is consistent with results reported in the existing literature. Waqas et al. suggest that empathy is a multidimensional construct, and the use of psychometric instruments designed to assess cognitive empathy may have led to different findings [38]. Healthcare and social care professionals with high scores on empathy scales may evaluate patients’ pain and desires regarding euthanasia through a cognitive process, considering others’ perspectives. In this context, their judgments may be influenced by their social and subjective norms, educational and professional backgrounds, and religious beliefs [38,39]. Religious beliefs, in particular, play a significant role in Greece and may substantially impact the cognitive process of adopting others’ perspectives. For instance, a study examining Greek healthcare professionals’ views on the acceptance of vaccination demonstrated the significant influence of religious issues on their attitudes and opinions [50]. We need to further explore how people make moral decisions. The role of the different forms of empathy should be further investigated. Although we can make some comparisons, the literature suggests that the mechanism of moral decision making about euthanasia is different for a suffering animal and a suffering human. It seems that people who are more empathetic are the most opposed to human euthanasia. However, other important parameters need to be explored, such as the individual’s ability to regulate emotions, the context in which a moral decision is made, and the type of euthanasia [51,52,53].

Additionally, our sample consists of diverse health and social care professionals, each of whom may interpret empathy differently due to their distinct scientific approaches and professional identities [54]. The cognitive approach to empathy varies among professionals. For instance, social workers and psychologists tend to adopt a psychosocial perspective on empathy, whereas doctors may approach it from a more paternalistic standpoint [54]. Consequently, their cognitive processes for imagining others’ perspectives are constructed in diverse ways.

## 5. Strengths and Limitations of This Study

Based on available evidence, this is the first report that examined the relationship of individual and professional characteristics, perceptions about death, and empathy with attitudes toward euthanasia in health professionals in Greece. The sample in this study includes a diverse range of healthcare professionals, which we regard as a key strength of our research design. Nevertheless, there is an overrepresentation of physicians—an outcome that is partly anticipated given the healthcare context—as well as social workers. Future studies may benefit from employing more homogeneous samples focused on a single professional group to allow for greater specificity and comparability. Although the cross-sectional design of this study limits causal inference, future researchers should also consider exploring associations of attitudes toward euthanasia with personal perception of death and empathy in health professionals working with end-stage patients only or with patients with a specific life-threatening illness. Furthermore, it is important to acknowledge that, while the manuscript detailing the validation of the ATE is currently under peer review and is expected to be published in the near future, it would have been preferable for it to have been available prior to the publication of the present study.

## 6. Conclusions

Euthanasia and assisted suicide continue to generate considerable debate within the public sphere. These practices present multifaceted moral, ethical, and legal dilemmas that resist straightforward resolution. For healthcare professionals, the complexities arising from competent patients’ requests for hastened death extend far beyond mere compliance with national legislation. Countries with legal frameworks and policies comparable to Greece’s may find it beneficial to explore the reasons behind the lack of open dialogue on euthanasia, particularly as the legalization of this practice is increasingly being discussed in numerous other nations. Finally, educating and supporting emerging generations of health professionals in patient care and bioethics are essential for equipping them to navigate the complex ethical challenges they will encounter throughout their clinical practice.

## Figures and Tables

**Table 1:** Demographic and professional characteristics of the healthcare personnel.

Variable		*n*	%
Gender	Male	117	25.2
	Female	348	74.8
Age (mean ± SD) (min–max)		42.6 ± 10.9	20–79
Age groups	≤30	74	15.9
	31–40	139	29.9
	41–50	140	30.1
	51+	112	24.1
Marital Status	Single	185	39.8
	Married	241	51.8
	Divorced	32	6.9
	Widowed	7	1.5
Educational level	Secondary Education	27	5.8
	University	236	50.8
	Master’s/PhD	202	43.4
Profession	Social workers	185	39.8
	Physicians	122	26.2
	Nurses	79	17.0
	Physiotherapists	31	6.7
	Other health professionals	21	4.5
	Psychologists	20	4.3
Professional experience	0–5	108	23.2
(years)	6–10	76	16.3
	11–15	71	15.3
	16–20	71	15.3
	21–30	92	19.8
	>31	47	10.1
Employment status	Permanent	303	65.1
	Not permanent	162	34.9
Working hours	Full time	451	97.0
	Part time	14	3.0
Sector	Public	313	67.3
	Private	152	32.7
Workplace	Hospital	266	57.2
	Other	199	42.8
Working with end-stage	Yes	256	55.1
Patients	No	209	44.9
Target group	Adults	222	47.7
	Elders	44	9.5
	Minors	34	7.3
	>1 target group	165	35.5

**Table 2:** Demographic effects on attitudes about euthanasia (ATE scale).

		ATE Scale			*p*
		*n*	Mean	SD	
Gender	Male	117	25.0	9.1	0.168 ^1^
	Female	348	23.8	9.2	
Age	≤30	74	22.3	7.2	0.293 ^2^
	31–40	139	24.2	8.4	
	41–50	140	24.0	9.6	
	51+	112	25.3	10.4	
Marital status	Single	185	23.5	8.3	0.213 ^2^
	Married	241	24.1	9.4	
	Divorced/Widowed	39	26.7	11.2	
Educational level	Secondary Education	27	22.9	8.3	0.889 ^2^
	University	236	24.1	9.0	
	Master’s/PhD	202	24.2	9.5	

^1^ Mann–Whitney; ^2^ Kruskal–Wallis.

**Table 3:** Professional effects on attitudes toward euthanasia (ATE scale).

		ATE Total			*p*
		*n*	Mean	SD	
Professional group *	Health professionals	201	25.0	9.8	0.155 ^2^
	Psychosocial/Mental health professionals	205	23.5	9.0	
	Other	59	22.9	6.9	
	health professionals	201	25.0	9.8	0.155 ^2^
Professional experience	0–5	108	22.3	8.2	0.360 ^2^
	6–10	76	24.2	8.6	
	11–15	71	24.4	9.4	
	16–20	71	24.4	8.3	
	21–30	92	25.4	10.8	
	>31	47	24.6	9.4	
Employment status	Permanent	303	24.3	9.3	0.594 ^1^
	Not permanent	162	23.7	8.8	
Working hours	Full time	451	24.1	9.2	0.989 ^1^
	Part time	14	24.0	9.1	
Sector	Public	313	24.6	9.3	0.110 ^1^
	Private	152	23.1	8.7	
Health service category and specialization	Non-specialized/General medical unit	411	24.3	9.2	0.263 ^2^
	Palliative care	34	21.8	9.9	
	Loss and bereavement management	14	25.1	7.6	
	Other	6	21.8	8.0	
Working with end-stage patients	Yes	256	23.6	9.5	0.251 ^1^
	No	209	24.7	8.7	
Target group	Adults	222	25.1	9.6	**0.045 ^2^**
	Elders	44	20.7	8.6	
	Minors	34	24.5	8.8	
	>1 target group	165	23.6	8.5	

^1^ Mann–Whitney; ^2^ Kruskal–Wallis; * Health Professionals: Physicians and Nurses; Psychosocial/Mental Health Professionals: Social workers and Psychologists.

**Table 4:** Correlation of ATE scale vs. DAP-R and JSE scales.

ATE	JSE	FoD	DaV	Nac	Aac	Eac
**R**	−0.008	0.010	**−0.358**	**0.099**	**0.092**	**−0.173**
**P**	0.856	0.829	**0.000**	**0.032**	**0.048**	**<0.001**

r: Spearman’s r; List of Abbreviations: FoD: Fear of Death, DaV: Death Avoidance, Nac: Neutral Acceptance, Aac: Approach Acceptance, Eac: Escape Acceptance.

## Data Availability

The data presented in this study are available on request from the corresponding author due to privacy and ethical restrictions.

## Supplementary Materials

The following supporting information can be downloaded at: https://www.mdpi.com/article/10.3390/healthcare13141731/s1, Figure S1: Self-referred religiosity of the participants; Figure S2: Correlation of ATE total score with DAP-R Death Avoidance (DAv) scale; Figure S3: Differences of ATE total score with Religiosity scale; Table S1: Responses of each ATE item; Table S2: Pearson’s and Spearman’s rho coefficients of ATE scale with DAP-R and JSE scale.
