# Supplementary material for: Health Professionals’ Views on Euthanasia: Impact of Traits, Religiosity, Death Perceptions, and Empathy
Source: Healthcare (Basel). 2025 Jul 18;13(14):1731. doi: 10.3390/healthcare13141731 (PMC12294693; doi:10.3390/healthcare13141731)
Supplement: Supplementary file 1 [file healthcare-13-01731-s001.zip › healthcare-3706088-supplementary.pdf]

Supplementary Material

Figure S1. Self-referred religiosity of the participants

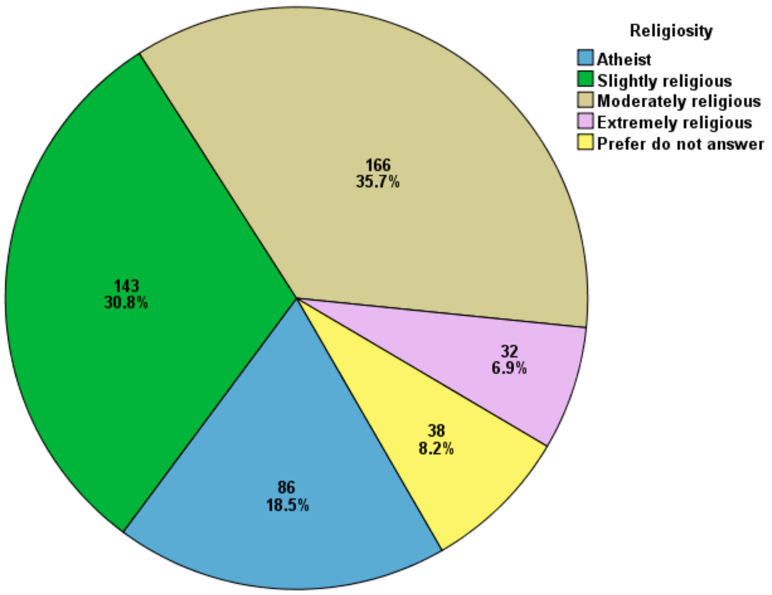

Figure S2. Correlation of ATE total score with DAP-R Death Avoidance (DAv) scale

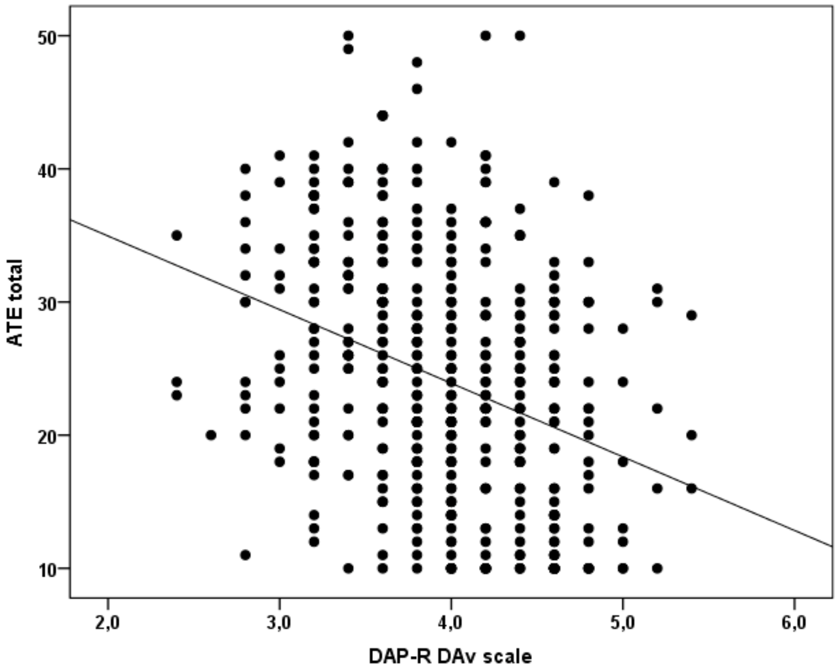

Figure S3. Differences of ATE total score with Religiosity scale

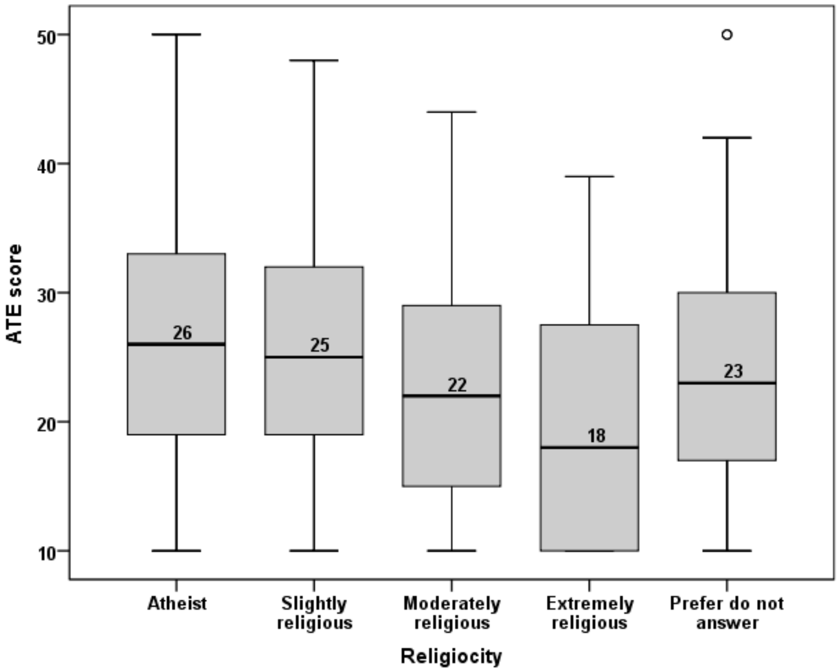

Table S1 Responses of each ATE item

	**Strongly disagree**		**Disagree**		**Undecided**		**Agree**		**Strongly agree**	
**ATE**	**n**	**%**	**n**	**%**	**N**	**%**	**n**	**%**	**n**	**%**
**ATE1**	90	19.4	72	15.5	**168**	**36.1**	95	20.4	40	8.6
**ATE2**	**230**	**49.5**	87	18.7	86	18.5	47	10.1	15	3.2
**ATE3**	**163**	**35.1**	85	18.3	129	27.7	64	13.8	24	5.2
**ATE4**	**200**	**43.0**	83	17.8	105	22.6	56	12.0	21	4.5
**ATE5**	**204**	**43.9**	108	23.2	88	18.9	49	10.5	16	3.4
**ATE6**	21	4.5	50	10.8	77	16.6	127	27.3	**190**	**40.9**
**ATE7**	**192**	**41.3**	102	21.9	98	21.1	54	11.6	19	4.1
**ATE8**	116	24.9	76	16.3	**122**	**26.2**	105	22.6	46	9.9
**ATE9**	51	11.0	69	14.8	101	21.7	103	22.2	**141**	**30.3**
**ATE10**	85	18.3	59	12.7	117	25.2	**127**	**27.3**	77	16.6

Table S2. Pearson's and Spearman's rho coefficients of ATE scale with DAP-R and JSE scale

**ATE**		**JSE**	**FoD**	**DaV**	**Nac**	**Aac**	**Eas**	**DAP-R**
**Pearson**	**R**	0.010	-0.004	**-0.338**	**0.113**	**0.095**	**-0.148**	0.055
	**P**	0.821	0.929	**0.000**	**0.015**	**0.040**	**0.001**	0.233
**Spearman**	**R**	-0.008	0.010	**-0.358**	**0.099**	**0.092**	**-0.173**	0.054
	**P**	0.856	0.829	**0.000**	**0.032**	**0.048**	**<0.0001**	0.244
